# Hand rehabilitation with sonification techniques in the subacute stage of stroke

**DOI:** 10.1038/s41598-021-86627-y

**Published:** 2021-03-31

**Authors:** Alfredo Raglio, Monica Panigazzi, Roberto Colombo, Marco Tramontano, Marco Iosa, Sara Mastrogiacomo, Paola Baiardi, Daniele Molteni, Eleonora Baldissarro, Chiara Imbriani, Chiara Imarisio, Laura Eretti, Mehrnaz Hamedani, Caterina Pistarini, Marcello Imbriani, Gian Luigi Mancardi, Carlo Caltagirone

**Affiliations:** 1 Istituti Clinici Scientifici Maugeri, I.R.C.C.S., Istituti Clinici Scientifici Maugeri, Music Therapy Research Laboratory, Scientific Institute of Pavia , Via Maugeri 10, 27100 Pavia, Italy; 2 Fondazione S. Lucia, I.R.C.C.S., Rome, Italy; 3 Istituti Clinici Scientifici Maugeri, I.R.C.C.S., Nervi (GE), Pavia, Italy; 4 Neurological Clinic, S. Martino Hospital, University of Genoa, Genoa, Italy; 5 Istituti Clinici Scientifici Maugeri, I.R.C.C.S, Montescano, PV Italy

**Keywords:** Health care, Neurology

## Abstract

After a stroke event, most survivors suffer from arm paresis, poor motor control and other disabilities that make activities of daily living difficult, severely affecting quality of life and personal independence. This randomized controlled trial aimed at evaluating the efficacy of a music-based sonification approach on upper limbs motor functions, quality of life and pain perceived during rehabilitation. The study involved 65 subacute stroke individuals during inpatient rehabilitation allocated into 2 groups which underwent usual care dayweek) respectively of standard upper extremity motor rehabilitation or upper extremity treatment with sonification techniques. The Fugl-Meyer Upper Extremity Scale, Box and Block Test and the Modified Ashworth Scale were used to perform motor assessment and the McGill Quality of Life-it and the Numerical Pain Rating Scale to assess quality of life and pain. The assessment was performed at baseline, after 2 weeks, at the end of treatment and at follow-up (1 month after the end of treatment). Total scores of the Fugl-Meyer Upper Extremity Scale (primary outcome measure) and hand and wrist sub scores, manual dexterity scores of the affected and unaffected limb in the Box and Block Test, pain scores of the Numerical Pain Rating Scale (secondary outcomes measures) significantly improved in the sonification group compared to the standard of care group (time*group interaction < 0.05). Our findings suggest that music-based sonification sessions can be considered an effective standardized intervention for the upper limb in subacute stroke rehabilitation.

## Introduction

After a stroke event, most survivors (80–90%) suffer from arm paresis, poor motor control and other disabilities, which evolve in a chronic condition in about 30–40% of cases^1,2^. Consequently, patients have difficulty in performing activities of daily living, a fact that severely affects their quality of life and independence^3^. Task-oriented sensory-motor training, which allows to transmit the sensory information of the feedback to the central nervous system during task execution, and movements adaptation, is recognized as significant in increasing post-stroke arm function and dexterity^2,4^. Beside other factors, such as patient’s compliance and subjective interest in stimulus, the intensity of training and patient’s motivation have been indicated as key features for a successful rehabilitation therapy^5,6^.

The development of technologies for rehabilitation has made it possible to formulate new application paradigms obtained by integrating the current rehabilitation pathways with instrumental interventions^7,8^. Sensory-motor rehabilitation techniques, obtained through technological devices (such as virtual reality, robots, non-invasive stimulations , motion capture) and used in support of traditional rehabilitation techniques, seem to provide objective parameters for patient evaluation, accelerate the process of motor recovery and improve motor performance at discharge by means of a top-down approach^9,10^.

Although the results obtained with the currently available devices are encouraging, we are only at an early stage for the exploitation of these technologies. In fact, while technology-assisted rehabilitation of the upper limb has demonstrated to have a significant impact on motor outcomes, especially at the proximal level (shoulder and elbow)^11^, the functional recovery of the hand (in particular of the prehension function) still presents some issues^12,13^.

Music and its elements (especially the rhythm) are widely used in rehabilitation^14^.This is due to the activation of the neuronal networks**,** in the limbic and paralimbic areas, and in the brain areas involved in movement (motor cortex, supplementary motor area, cerebellum, basal ganglia, etc.)^15,16^. Many studies document the possibility that exposure to music during training, but, also through specific rehabilitation interventions, may induce plastic changes^15–19^ in the sensory-auditory circuits and motor areas^20,21^. These changes, resulting in a neuronal reorganization in the nodal points of the brain networks and in fiber bundles, can determine effects lasting beyond the actual duration of the rehabilitation intervention^15^. Moreover, music in the rehabilitation process determines an emotional involvement, creating a strong motivational basis^15,16^.

The recent literature shows how the use of music in stroke rehabilitation can improve gait (speed, cadence and step length, balance)^22,23^, movement of the upper limbs^24,25^, language^26,27^, but also mood and other psychological aspects^28–30^. More recent studies use, for the rehabilitation of the upper limbs, the mapping of the patient’s movements to whom a sonorous stimulus is associated (sonification)^31–34^. Sonification can improve motor functions rehabilitation and can facilitate the integration of auditory and sensory-motor systems^35–37^. This technique can also strengthen and support the damaged proprioceptive system and can make the rehabilitative process more pleasant and stimulating from an emotional and motivational point of view^15,37^. In some studies, the kinematic data of the gesture are translated into modulations of some parameters (typically frequency and amplitude) of a continuous synthetic sound^38–40^.

In other post-stroke hand rehabilitation studies, the sonification action was based on the execution of short scales or melodic intervals that the patient had to reproduce through specific movements, with the final goal of creating "simple nursery rhymes or other familiar tunes", modulating music timbre and intensity^41^. Further, Reh et al.^42^ supported the rehabilitation of walking by a combination of an ecological sound (noise produced by a step in the snow) together with a predetermined glissando effect.

In this study the sonification can be considered as a properly selected set of sonorous-music stimuli activated by patient’s movements with the mediation of a sensor (in this specific case the Leap Motion Controller). Synthesized sounds/musical texture and their parameters (mainly rhythm, pitch/melody, intensity/dynamics, harmony and timbre) are used to represent movements characteristics, especially from a temporal and spatial point of view. Sonification makes possible the improvement of sensorimotor learning, proprioception, movements planning and execution^37^.

In particular music elements used in this study were automatically associated to the patient’s movements without involvement of any cognitive tasks. This allows the patient to focus attention mainly on the motor outcome. In particular, given the importance of hand functions in activities of daily living and manipulation, the protocol (Sonichand) was developed and validated for the training of pronation and supination of the forearm, ulnar and radial deviation of the wrist and hand grasping movements^43^.

The main objective of this randomized controlled study conducted in subacute stroke patients during inpatient rehabilitation, was to verify the effectiveness (evaluated through the assessment of the upper limb level of impairment at baseline and end of treatment) of a new rehabilitative hand treatment based on a musical sonification approach compared to a conventional intervention. The secondary aim was to evaluate if this technique could be beneficial for reduction of pain perceived during training and improve the patient's perceived quality of life. The main hypothesis of this study was that our sonification approach could be more effective than a conventional intervention for the rehabilitation of subacute stroke patients, especially for the recovery of hand motor function.

## Results

**Figure 1:**
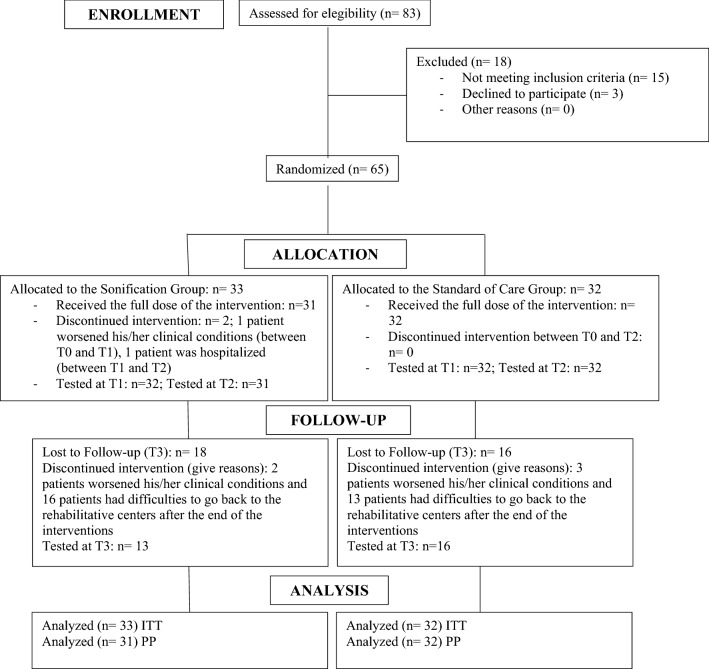
The flow chart of the study. All randomized patients received the allocated intervention. Between baseline and the 4-week evaluation two patients in the Sonification group discontinued the treatment. No discontinuation occurred in the Standard Care Group. After 1 month from the beginning of treatment (T3 follow-up) 18 and 16 patients were lost to follow-up in the Sonification and Standard Care Groups, respectively. The primary analysis (T2, end of the 4-week treatment) was performed on the Intention To Treat (ITT) and Per Protocol (PP) populations, with the numbers reported in the figure.

Clinical characteristics of the sample at baseline are reported in Table 1. Two patients allocated to the Sonification Group interrupted the trial between T0 and T2: the first one worsened his/her clinical condition and the second one was hospitalized. In addition, 18 patients in the Sonification Group and 16 patients in the Standard of Care Group were lost to follow-up due to the clinical worsening (2 patients in the Sonification Group and 3 patients in the Standard of Care Group) or to the difficulty to go back to the rehabilitative centers after the end of the interventions (16 patients in the Sonification Group and 13 patients in the Standard of Care Group) (Flow Diagram is reported in Fig. 1). No side effects were observed in both the experimental and control groups after training.

**Table 1:** Baseline clinical characteristics of patients in Sonification Group (SG) and Standard of Care Group (SoCG).

	SG (n = 33)	SoCG (n = 32)	p
	Mean ± SD or N (%)	Mean ± SD or N (%)	
Sex (males/females)	17 (51.5%) /16 (48.5%)	18 (56.2%) /14 (46.8%)	0.70^a^
Age (years)	62.4 ± 8.9	64.7 ± 16.0	0.52^b^
Handedness (right handed)	31 (93.9%)	31 (93.8%)	0.97^a^
**Acute event (days)**			0.68^c^
Median	34.5	29.5	
Interquartile Range	38.25	31.75	
Min–Max	12–180	13–180	
**Lesion side**			0.063^a^
Right	22 (66.7)	14(43.8)	
Left	11 (33.3)	18 (56.3)	

^a^Chi-square test; ^b^Student’s t test; ^c^Mann–Whitney U Test.

### Primary outcome

**Figure 2:**
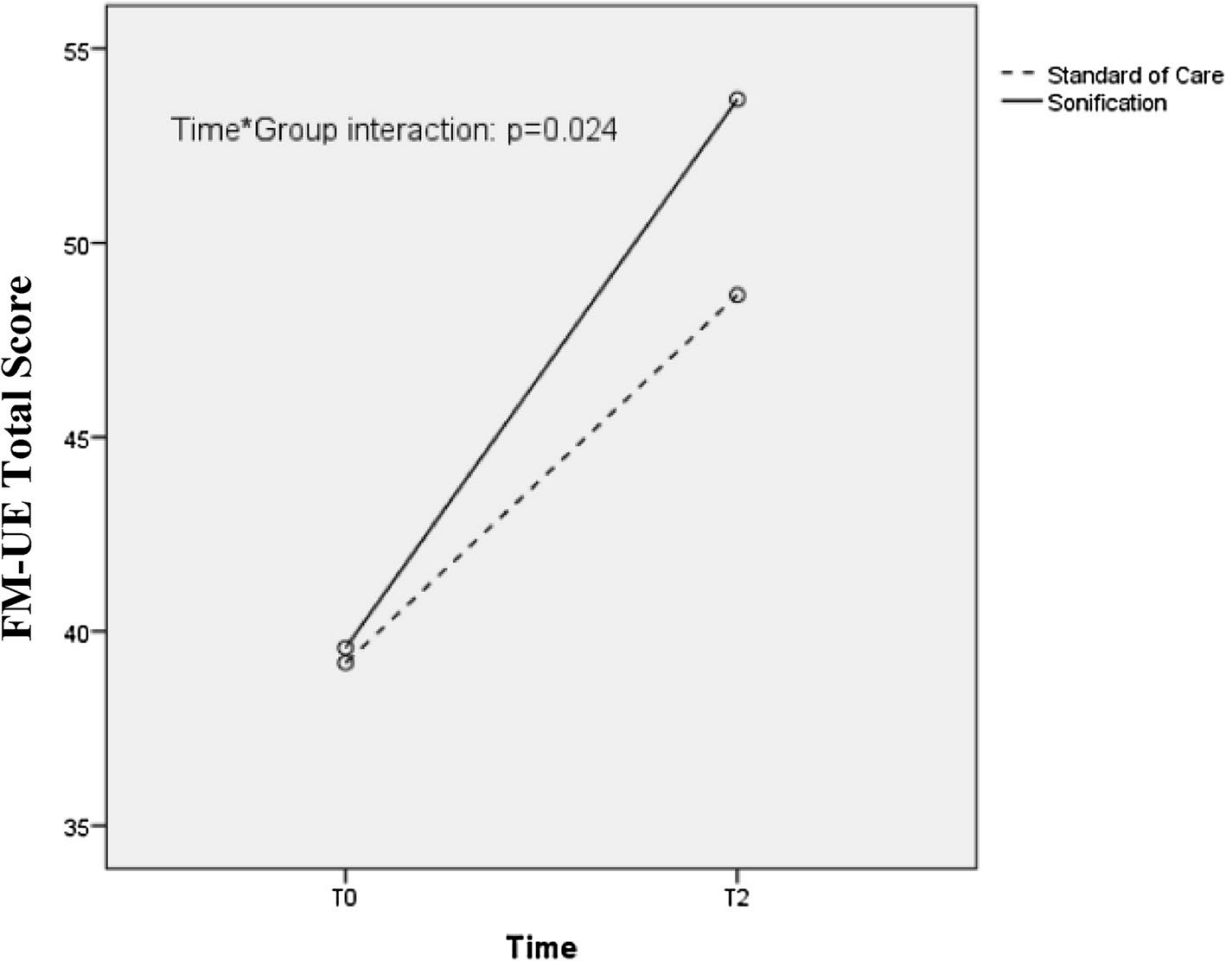
Trend between baseline (T0) and end of treatment (T2, 4 weeks) of primary outcome, FM-UE Total Score.

The upper limb impairment assessed by the Fugl-Meyer Upper Extremity scale total score^44^ , showed a significantly greater improvement between T0 and T2 (Table 2, Fig. 2) in the Sonification Group compared to the Standard of Care Group (time*group interaction: p = 0.024), as pointed out by the Intention To Treat analysis.

**Table 2:** Upper extremity scores (primary and secondary outcomes) at T0 and T2 (intention to treat analysis).

	SG (n = 33)	SoCG (n = 32)	Time*group interaction			Effect size	Time effect			Effect size	Group effect			Effect size
			F	df	p value		F	df	p value		F	df	p value	
**Primary outcomes**														
FM-UE Total score														
T0	39.58 ± 10.99	39.19 ± 14.94	5.326	1	**0.024**	0.078	136.93	1	** < 0.001**	0.685	0.665	1	0.418	0.010
T2	53.70 ± 12.03	48.66 ± 17.37												
**Secondary outcomes**														
FM-UE proximal score														
T0	22.27 ± 6.49	21.31 ± 9.09	1.424	1	0.237	0.022	99.56	1	** < 0.001**	0.612	0.884	1	0.350	0.014
T2	29.89 ± 6.23	27.31 ± 9.92												
FM-UE distal score														
T0	13.88 ± 4.81	14.12 ± 6.68	6.072	1	**0.016**	0.088	110.97	1	** < 0.001**	0.638	0.376	1	0.542	0.006
T2	19.82 ± 4.59	17.81 ± 7.71												
FM-UE wrist score														
T0	5.55 ± 2.11	5.78 ± 2.81	4.778	1	**0.033**	0.071	75.06	1	** < 0.001**	0.544	0.138	1	0.711	0.002
T2	7.85 ± 2.09	7.16 ± 3.29												
FM-UE hand score														
T0	8.33 ± 3.30	8.34 ± 4.13	4.391	1	**0.040**	0.065	88.67	1	** < 0.001**	0.585	0.538	1	0.466	0.008
T2	11.97 ± 2.98	10.66 ± 4.60												
BBT affected limb														
T0	13.39 ± 9.24	16.50 ± 14.27	5.657	1	**0.020**	0.082	87.65	1	** < 0.001**	0.582	0.137	1	0.712	0.002
T2	23.06 ± 10.80	22.25 ± 16.32												
BBT unaffected limb														
T0	32.12 ± 9.94	30.56 ± 12.06	8.873	1	**0.004**	0.123	42.87	1	** < 0.001**	0.405	2.530	1	0.117	0.039
T2	39.88 ± 9.63	33.47 ± 10.72												
BBT ratio														
T0	0.42 ± 0.25	0.58 ± 0.55	1.787	1	0.186	0.028	17.581	1	** < 0.001**	0.218	1.887	1	0.174	0.029
T2	0.57 ± 0.22	0.66 ± 0.45												
NPRS														
T0	3.79 ± 3.02	1.76 ± 2.44	7.972	1	0.006	0.112	12.46	1	0.001	0.165	4.120	1	0.047	0.061
T2	1.65 ± 2.23	1.52 ± 2.34												
MAS Wrist (a)														
T0	0 (1)	0 (1)	2.46	1	0.117	–	97.0	1	0.186	–	0.302	1	0.583	–
T2	0 (1)	0 (1)												
MAS fingers (a)														
T0	0 (1)	0 (1)	2.534	1	0.111	–	13.5	1	**0.004**	–	0.391	1	0.532	–
T2	0 (0.5)	0 (0.75)												
MQoL-it														
T0	6.60 ± 1.41	6.89 ± 1.32	0.495	1	0.484	0.008	13.56	1	** < 0.001**	0.177	0.373	1	0.543	0.006
T2	7.32 ± 1.34	7.37 ± 1.06												

Values for continuous variables are reported as mean ± standard deviation, those for categorical data are reported as median and interquartile range.

*SG* Sonification Group, *SoCG* Standard of Care Group, *FM-UE* Fugl-Meyer Upper Extremity scale, *BBT* Box and Block Test, *NPRS* Numerical Pain Rating Scale, *MAS* Modified Ashworth Scale, *MQoL-it* McGill Quality of Life (Italian Version) , *df* degrees of freedom; (a) = non-parametric (Kruskall-Wallis) statistics.

### Secondary outcomes

**Figure 3:**
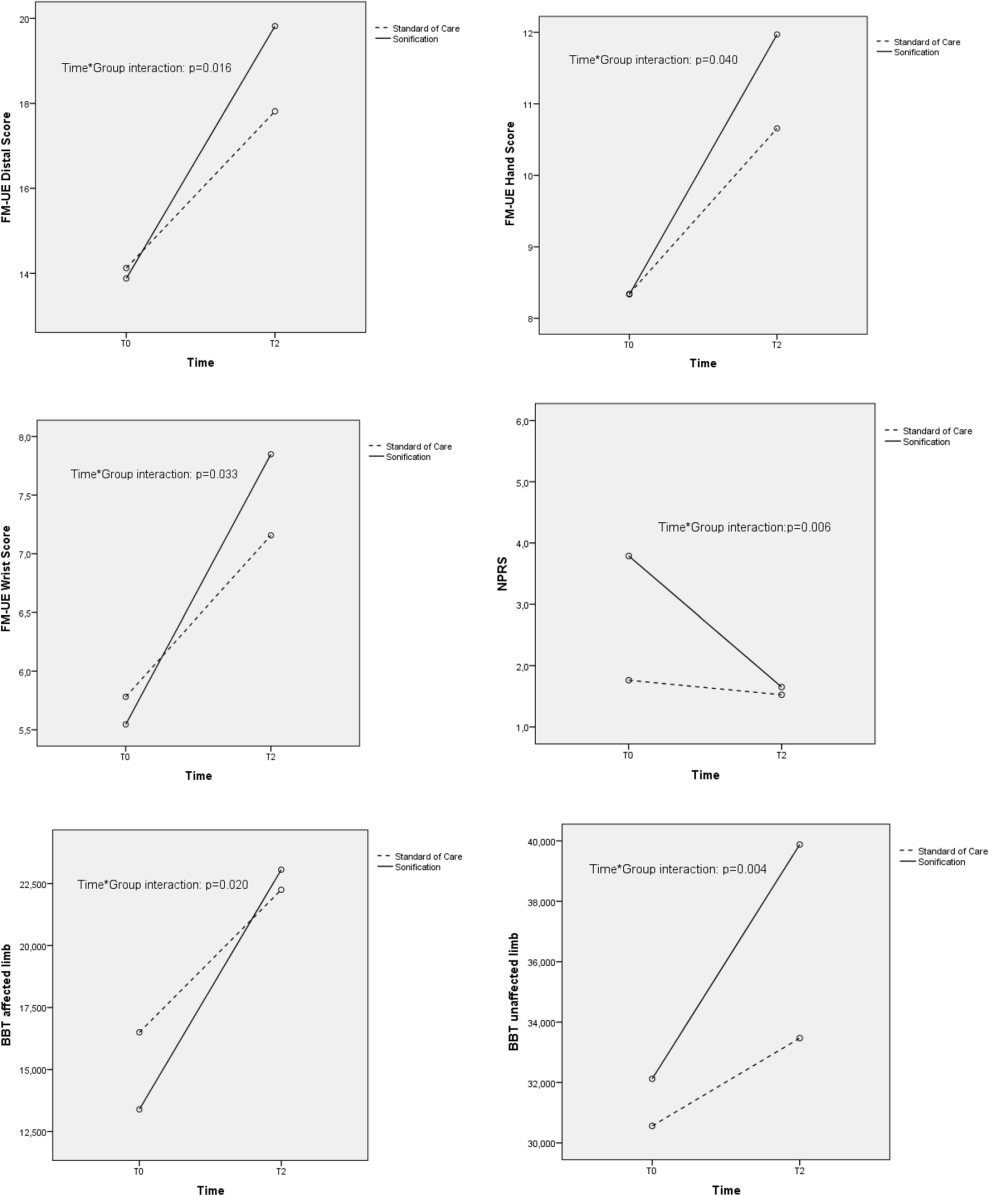
Trend between baseline (T0) and end of treatment (T2, 4 weeks) of secondary outcomes.

At the Intention To Treat analysis, other significantly greater improvements between T0 and T2 in the Sonification versus the Standard of Care Groups were found for the Distal Score (time*group interaction: p = 0.016), Wrist Score (time*group interaction: p = 0.033) and Hand Score (time*group interaction: p = 0.04) subscales. Also, Manual dexterity significantly improved (T0 vs T2) in the Sonification Group as showed by the Box and Block Test^45^ (affected limb time*group interaction: p = 0.020; unaffected limb time*group interaction: p = 0.004). The Box and Block Test score Ratio improvement was not significant (time*group interaction: p = 0.19). In addition to motor outcomes the study put in evidence the effects of sonification in pain perception: the Sonification Group showed a significant reduction in Numerical Pain Rating Scale^46^ scores compared to the Standard of Care Group (time*group interaction: p = 0.006). No significant changes were observed in spasticity (Modified Ashworth Scale)^47^ and perceived quality of life (McGill Quality of Life)^48^. All results are summarized in Table 2 and Fig. 3 and the same findings were confirmed at the Per Protocol analyses (Supplementary Table S1).

## Discussion

The study confirmed how the sonification with music elements is able to facilitate the recovery process compared to the traditional rehabilitative approach. Main results are related to the global upper limb impairment (primary endpoint of the study), assessed by the Fugl-Meyer Upper Extremity scale (FM-UE). In particular, both the experimental (SG) and control (SoCG) groups scored a clinically significant increase of the FM-UE total score after training, but only the SG had a clinically significant (> 5) improvement^49^. Conversely to the control group the experimental group did not show a significant improvement at proximal level at T2. A possible explanation could be that the Sonichand protocol involves mainly the distal segments of the arm and this aspect should be considered in future studies. Results of the present study can be extended to manual dexterity, as testified by the improved Box and Block Test (BBT), both in affected and unaffected limbs. The score improvement observed for the presumed-to-be-unaffected upper extremity could be due to a learning phenomenon obtained during repeated evaluations or to undetected motor deficit due to the brain lesion thus involving slight abnormal kinematics subject to improvements^50^. In addition, some BBT variability could be due to a day-by-day effect that however should be minimized by taking into consideration the ratio score^51^.

In accordance with Kinney et al.^52^ thresholds, our study showed a large effect size for time effect (> 0.55) for both FM-UE total and subscales values and for the BBT of the affected upper limb, a medium effect size (≥ 0.31, < 0.55) for BBT of unaffected upper limb and a small effect size (≥ 0.14, < 0.31) for the remaining outcome variables. Conversely to a study for robot assisted hand training^53^, the effect size for group effect of our study was not statistically significant for all outcome variables. This could be likely due to the different training device and intensity of training (20 min. vs. 60 min.) adopted in the two studies. Finally, small effect sizes of the time*group interactions should be evaluated in a framework of power of interactions in the analysis of variance models.

The sonification technique used in our study was very intuitive, compared to other similar interventions^37^. In fact, music support was intrinsically associated with the patient's movements, regardless of his/her intention to produce sounds. This peculiarity allowed the patient to focus mainly on the movement and, at the same time, to receive a natural feedback on its coherence and quality (i.e. a correct movement produced a fluent musical progression). The above mentioned mechanisms could have helped the patient to model/adjust and re-learn movements during the rehabilitative intervention. Another important result was found by evaluating the effect of sonification on pain perception associated to upper extremity movements during the rehabilitation intervention. Despite higher levels of perceived pain (Baseline scores) in the Sonification group compared to those perceived by the control group patients, the music-based rehabilitative approach used in this study was able to produce a significant decrease of NPRS score. In accordance with the literature concerning the use of music in pain medicine, our data showed how the presence of musical patterns can significantly reduce the perceived pain, likely because music helps patients to get distracted from the pain condition^54^.

The harmonic and melodic progressions used in the study, compared to other possible sounds (e.g. sinusoidal tones) could have contributed to create predictable and pleasant sequences, involving the recall and recognition of the tonal trend typical of the music listening experience. In addition, the predictability of sequences could also have produced a facilitation effect during the preparation and execution of movements.

One of the main limitations of the study is the limited data available for the planned follow-up assessment at 1 month after the end of treatment (T3). In spite of therapists’ recommendation, only less than 50% of patients completed the study, making the reliability of results at T3 questionable. This could be due to the patients’ difficulties to get back to the rehabilitative centers after the end of the interventions and/or to other reasons, such as a lack of motivation/unwillingness due to a follow-up too close to the study conclusion and conflicting with the new difficulties of daily life activities of the whole family.

For this reason, we decided to include the follow-up analysis only into the supplementary materials (Supplementary Table S2). This analysis showed a maintenance of results over time. Another limitation of the study is that we did not apply block randomization in the allocation of patients to experimental and control groups. Taking into account the defined exclusion criteria, the block randomization would not have guaranteed in a reasonable time, patients’ enrollment at the different Units, with a balanced and homogeneous sample size. In addition, an increased sample size could have produced greater effect size to support the beneficial effect of the sonification technique.

Finally, the lack of other evaluation methods, such as neuroimaging techniques (fMRI) aimed at pointing out possible changes in the brain connectivity and their possible correlation with post-treatment performance indexes, should be considered as a further limitation.

The study proved that this type of music intervention can improve movements and reduce pain during the rehabilitation process in patients with stroke. Upper limb rehabilitation with sonification techniques can be considered a standardized intervention, effective and easy to implement in subacute stroke rehabilitation. The low-cost of the sonification set-up makes the intervention widely exploitable into the standard occupational/physical therapy settings. Also, it is expected that the Sonichand protocol and approach, thanks to the powerful feedback produced by movements sonification, could be beneficial also for the treatment of other neurological conditions in which upper limbs movements are impaired due to specific brain lesions (i.e., gesture disturbances, apraxia and poor gesture imitation). Future studies involving other clinical conditions and with a more comprehensive assessment protocol, could strengthen the significant findings obtained by this study.

## Methods

This randomized controlled trial involved 65 subacute stroke patients who underwent inpatient rehabilitation allocated into two groups: the SG (n = 33) underwent sonification sessions (see “Interventions” sub-section) and the SoCG (n = 32) underwent traditional motor rehabilitation sessions. The randomization was centrally managed: a randomization list (simple randomization), generated accordance with a completely randomized parallel group study design, was generated at the beginning of the study and delivered to the Principal Investigator (PI) of the study. Each recruited subject was allocated in SG or SoCG by the PI on the basis of the randomization list by assigning a unique identifying code so to guarantee data anonymization during data collection, analysis and interpretation processes. The blindness of the evaluations was guaranteed both for questionnaires data collection and statistical processing.

Subjects were recruited from March 27th 2017 to April 15th 2019 in 5 Italian rehabilitative units located at Maugeri Scientific Clinical Institutes of Pavia, Montescano (PV) and Nervi (GE), S. Lucia Foundation IRCCS (Rome) and Neurological Clinic of S. Martino Hospital (University of Genoa). All procedures performed in this study were in accordance with the ethical standards of the institutional and/or national research committee and with the 1964 Helsinki declaration and its later amendments or comparable ethical standards. The study was approved by the Ethical Board of the Istituti Clinici Scientifici Maugeri IRCCS, Pavia, Italy (2088 CE, December 19th, 2016) and registered on https://clinicaltrials.gov (Trial Number Identifier: NCT03306797, date of registration: 11/10/2017), all participants gave their written informed consent to participate in the study.

### Inclusion and exclusion criteria

Patients matching the following characteristics were included in the study:

- Age 40–85 years
- Ischaemic lesion in a single hemisphere (right or left hemiplegia/hemiparesis)
- Clinically evaluable residual movement capacity of the paretic upper limb (ability to autonomously make postural adjustments during reaching tasks)
- Mini Mental State Examination > 24
- Acute onset no more than 180 days prior to enrollment in the study

The exclusion criteria of the Study were the following:

- Presence of neglect (evaluated through a clinical and functional assessment)
- previous or concomitant diseases affecting upper limb functions (e.g. Parkinson's disease, multiple sclerosis, shoulder periarthritis, Dupuytren's disease, etc.)
- rehabilitation treatments with music in the last year

### Interventions

The patients recruited in the study were admitted to the rehabilitation centers and received usual treatment for their subacute stage of disease (such as occupational therapy, speech therapy, psychological support, lower extremity rehabilitation, etc.) in addition to a specific upper extremity rehabilitation based on (a) standard motor exercises or (b) standard motor exercises supported by sonification techniques.

#### Standard motor exercises

The upper limb rehabilitation was carried out in accordance with the Italian Stroke Prevention and Educational Awareness Diffusion (SPREAD) guidelines^55^ and took into account a traditional neuro-motor approach aimed at improving motor control and reducing spasticity. At the same time, these methods were combined with an individualized and progressive functional re-education through task-oriented programs^56^. The first phase of the treatment (passive treatment, 15 min) aimed at relaxing muscle tone with stretching. The exercises were chosen, based on the needs and residual motor skills of the patient, from among the following:

- fingers opening and closing
- prono-supination of the forearm
- wrist mobilization

The second phase of the treatment (20 min) included movements actively performed by the patient. The patient was offered at least 6 different exercises. Each exercise lasted 90 s and was followed by a break of 30–60 s. The exercises were chosen from the following:

1. Forearm–wrist: (a) flexion and extension of the wrist in the intermediate position of the forearm (horizontal plane), (b) flexion and extension of the wrist with pronated arm (vertical plane), (c) ulnar and radial deviation of the wrist, (d) pronation and supination of the forearm.
2. Hand: (a) grasp; (b) pinch; (c) finger extension (d) abduction and adduction of the fingers
3. Shoulder-elbow: (a) elbow flexion and extension; (b) combined movement of shoulder flexion and elbow extension

During the active phase the therapist assisted the patient in the movement that took place autonomously, with the active participation of the patient. The exploration of the workspace was carried out using tools such as a table facilitating sliding of the arm on the work surface. If compatible with patient’s impairment and performance, grip-release exercises of medium-volume objects and their displacement in the space were also included.

#### Standard motor exercises supported by sonification techniques

This intervention was similar to the above mentioned treatment (a), i.e. it included a passive treatment phase without sonification (15 min) and a second phase (20 min) in which the motor exercises were supported by sonification techniques. In this second phase of treatment the patient performed the movements actively, by practicing a sequence of exercises lasting about 90 s each, separated by a 30–60 s resting period.

The sonification process was made using an adequate setup including a technological sensor (Leap Motion Controller)^57–59^, managed with an ad-hoc developed application parameterizing the data read by the sensor by producing sounds in response to some specific hand movements. The Leap Motion Controller (LMC; Leap Motion, Inc., San Francisco, CA, USA) is described as a low-cost Human Computer Interface specifically optimized for the recognition of gestures produced by hands, fingers and pointer objects, with a precision, validated in real situation, of about 0.7 mm. The LMC is connected to the computer through a USB 3.0 communication interface and does not require contact with the patient. Thanks to the use of 3 infrared LEDs and 2 optical sensors, it can create a three-dimensional workspace of about 120 cm × 60 cm × 60 cm. The LMC is capable to capture the movement of hands and fingers thanks to two monochromatic cameras and three infrared LEDs and it is able to build a vector representation of the hand, fingers, wrist and forearm up to the elbow. The device is not so precise for temporal responses but, unlike tapping, this type of exercises do not need a strict temporal association between movements and sounds. Before starting, the minimum and maximum points of the movements (range of motion) are measured. If the sonification is discrete (e.g. 4 notes of a musical scale) this range is further divided into thresholds. During the performance, if the read value falls within a preset range around a minimum/maximum threshold, a preset sound sample is played (basically each threshold is a trigger for the assigned note). If the sonification is continuous, the read value will modulate the sound along a continuum, from the minimum to the maximum level measured (range of motion). In this trial we used a sampling rate of 25 Hz.

The sensor, which is positioned under the palm of the hand at a distance of about 15 cm, identifies and maps the limb, creating a representation of it in points, providing the relative kinetic characteristics (spatial coordinates, acceleration, direction, inclination, etc.) for each point. The software program (https://ismm.ircam.fr/leapmotion/ ), used to carry out our experiments, was developed with the Max 7 software programming environment (Max 7 by Cycling ’74, Walnut, CA, USA, https://cycling74.com). Thanks to this application the patient's gestures are supported by two modalities:

1. In the first, movements (forearm-wrist: flexion and extension of the wrist in the intermediate position of the forearm—horizontal plane-, flexion and extension of the wrist with pronated arm—vertical plane-, ulnar and radial deviation of the wrist; hand: pinch, finger extension; shoulder-elbow: elbow flexion and extension) trigger and modulate a harmonic progression built on the consecutive grades of the major scale played as an ascending and descending arpeggio also with a consistent volume (ascending crescendo and descending diminuendo sounds) (Supplementary Video S3).
2. In the second, movements (forearm-wrist: pronation and supination of the forearm; hand: grasp, abduction and adduction of the fingers; shoulder-elbow: combined movement of shoulder flexion and elbow extension) modulate the volume and low-pass cutoff frequency of a tone filter applied to a synthetic texture. It is a kind of tone modulation of a pad sound, from dull to bright, based on a harmonic progression built on the consecutive grades of a major scale (Supplementary Video S4).

On the basis of the exercises selected for rehabilitation, some music parameters (pitch, volume and spectrum—quality/brilliance of tone) have been identified and superimposed on the gesture.

The correct execution of the gesture leads patients to cyclically perform a harmonic progression of arpeggios. Considering the daily administration of the training protocol, different instruments were associated to the various exercises to increase the sound variability (for example: trumpet, flute, cello, guitar, piano etc., as well as some timbre variants for the pad performed by a synthesizer).

To ensure correct understanding of the training protocol, before the experimental intervention, the therapist measured the active range of motion for sensor mapping and provided a short demonstration of the exercises (learning phase) to the patient who carried out a short training with the non-plegic limb.

In addition, in order to guarantee uniformity of intervention, the experimental set up (including a wheelchair with a transparent table and a clamp to support the Leap Motion Controller) was replicated in each Unit involved in the study. All interventions (5 days a week for 4 weeks, for a total of 20 sessions) were assisted by physiotherapists or occupational therapists with an expertise in hand rehabilitation or neurorehabilitation and specifically trained to use the Leap Motion Controller and the sonification described setting. The therapists involved into the protocol received at least 8 h of specific training before the beginning of experiments.

### Assessment

The rehabilitation interventions were evaluated by blinded evaluators at baseline (T0), after 2 weeks (T1), at the end of treatment (T2, after 4 weeks) and 1 month after the end of the treatment (T3).

The FM-UE^44^ (score range 0–66) was used to evaluate the performance-based sensorimotor functions of the impaired upper limb. Furthermore, in order to detail the motor improvement both at the proximal and distal level, the FM-UE was sub-scaled into proximal (shoulder/elbow, score range 0–42), distal (wrist/hand, score range 0–24), wrist (score range 0–10) and hand (score range 0–14) subscores^60^. The Modified Ashworth Scale (MAS, score range 0–4; 6 categories) was used to evaluate muscle tone at the upper limb^61^. In accordance with previous studies, the score of ‘1+’ category was converted to 1.4 to be managed in statistical analysis^60^. The BBT was used as a measure of gross manual dexterity^62^. In particular, for this test we assessed the performance (number of blocks) both of the affected and unaffected limbs and their score ratio (BBT ratio = BBT affected limb/BBT unaffected limb) in order to compensate for their variability^51^.

Pain and quality of life assessment were rated using respectively the NPRS^63^ (score range 0–10) and the McGill Quality of Life (MQOL-it, score range 0–160)^48^.

### Outcomes measures

The primary outcome measure of the study was the evaluation of the improvement in total scores of the FM-UE between T0 and T2. Secondary outcomes measure were other motor aspects (including scores of BBT, MAS and sub-items scores of the FM-UE), pain (scores of NPRS) and the perceived quality of life (MQOL-it).

### Statistics

Sample size: the sample size estimate is based on previous data acquired from results of rehabilitation interventions, with and without technology-assisted techniques, carried out at the facilities participating in the study^64^. For the sizing of the present study it is assumed that the use of a technological system for sonification allows to achieve at least clinically relevant results as traditional rehabilitation. An increase of at least 6 points in FM-UE is recognized to be the minimal clinically important difference between baseline and end of treatment following rehabilitation^54^. The work hypothesis, based on previous data, was that the pre-post treatment difference in FM-UE was at least 5 point higher in the SG than in the SoCG groups. With these hypotheses and assuming a standard deviation of the pre-post treatment differences of 10, a total sample of 65 subjects can guarantee a study power of 80% and a type I error of 5%.

### Data analysis

Descriptive statistics were carried out for all the collected variables. Means and standard deviations were reported for normally distributed quantitative variables. In case of significant discrepancy from the normality, medians and interquartile ranges were also included. Nominal level variables were summarized as frequencies and percentages. Baseline clinical characteristics between SG and SoG groups were compared by means of Student’s t test, Mann–Whitney U test or chi-square test according to the nature and distribution of the variables. Inferential statistics were performed on the Intention To Treat population defined as all randomized subjects. Handling of missing data was managed according to a strategy pre-specified in the study protocol and referred to the four time points of assessment. Imputation strategy included the handling of missing at random and not at random. Generalized linear mixed models were applied to handle missingness at single timepoints where measured repeatedly over time. Last Observation Carried Forward (LOCF) method was applied to missingness of patients who prematurely interrupted the trial. Analyses on the PP population were also conducted to support the findings.

Repeated measures analysis of variance with one factor (factor: experimental/control group) or non-parametric approach (Kruskall-Wallis statistics) was used to assess potential differences between baseline (T0) and end of treatment (T2) for the primary and secondary endpoints. The same models were applied to assess trends over time at the four assessment timepoints foreseen in the protocol (T0 = baseline T1 = 2 weeks of treatment, T2 = 4 weeks, end of treatment, T3 = 8 weeks, follow-up). Due to the huge amount of missing data at the follow-up (T3), this analysis was carried out on the per protocol population only without any imputation of missing data. Effect sizes of the main effects, repeated measures and interactions were finally computed to point out the magnitude of the effects in a standardized metric with the aim of favoring comparisons with other studies. Effect size was rated as small (d = 0.14), medium (d = 0.31) and large (d = 0.55) in accordance with the effect size interpretation guidelines provided by Kinney and coll. for a study classified as a Multicomponent intervention^52^. Experiment-wise error rate was set at 5%. All analyses were conducted using SPSS Statistics Professional package.

## Supplementary Information

Supplementary Information 1.

Supplementary Information 2.

Supplementary Video 1.

Supplementary Video 2.

## Data Availability

The datasets generated during and/or analyzed during the current study are available from the corresponding author on reasonable request.
