# Supplementary material for: Hand rehabilitation with sonification techniques in the subacute stage of stroke
Source: Sci Rep. 2021 Mar 31;11:7237. doi: 10.1038/s41598-021-86627-y (PMC8012636; doi:10.1038/s41598-021-86627-y)
Supplement: Supplementary file 1 — Supplementary Information 1. [file 41598_2021_86627_MOESM1_ESM.docx]

**Supplementary Table S1.** Upper Extremity Scores (Primary and secondary outcomes) at T0 and T2 of the per protocol analysis. Values for continuous variables are reported as mean ± standard deviation, those for categorical data are reported as median and interquartile range.SG= Sonification Group; SoCG= Standard of Care Group; FM-UE= Fugl-Meyer Upper Extremity scale; BBT= Box and Block Test; NPRS= Numerical Pain Rating Scale; MAS= Modified Ashworth Scale; MQoL-it= McGill Quality of Life (Italian Version); df=degrees of freedom; (a)=non-parametric (Kruskall-Wallis) statistics.

|  | SG  (n=31) | SoCG  (n=32) | Time*group interaction | | | Effect Size | Time effect | | | Effect Size | Group effect | | | Effect Size |
| --- | --- | --- | --- | --- | --- | --- | --- | --- | --- | --- | --- | --- | --- | --- |
| **Primary Outcomes** |  |  | F | df | P-value |  | F | df | P-value |  | F | df | P-value |  |
| FM-UE Total Score  T0  T2 | 39.75 ± 11.12  54.31 ± 11.68 | 39.19 ± 14.94  48.66 ± 17.37 | 6.510 | 1 | **0.013** | 0.095 | 144.89 | 1 | **<0.001** | 0.700 | 0.858 | 1 | 0.358 | 0.014 |
| **Secondary Outcomes** |  |  |  |  |  |  |  |  |  |  |  |  |  |  |
| FM-UE Proximal Score  T0  T2 | 21.93 ± 6.56  30.10 ± 6.40 | 21.31 ± 9.09  27.31 ± 9.92 | 2.444 | 1 | 0.123 | 0.039 | 104.50 | 1 | **<0.001** | 0.635 | 0.754 | 1 | 0.389 | 0.012 |
| FM-UE Distal Score  T0  T2 | 13.97 ± 4.86  20.09 ± 4.38 | 14.12 ± 6.68  17.81 ± 7.71 | 7.202 | 1 | **0.009** | 0.104 | 116.71 | 1 | **<0.001** | 0.653 | 0.540 | 1 | 0.465 | 0.009 |
| FM-UE Wrist Score  T0  T2 | 5.56 ± 2.14  7.94 ± 2.06 | 5.78 ± 2.81  7.16 ± 3.29 | 5.542 | 1 | **0.022** | 0.082 | 77.93 | 1 | **<0.001** | 0.557 | 0.205 | 1 | 0.652 | 0.003 |
| FM-UE Hand Score  T0  T2 | 8.41 ± 3.33  12.16 ± 2.83 | 8.34 ± 4.13  10.66 ± 4.60 | 5.191 | 1 | **0.026** | 0.077 | 92.33 | 1 | **<0.001** | 0.598 | 0.766 | 1 | 0.385 | 0.012 |
| BBT affected limb  T0  T2 | 12.53 ± 9.41  22.71 ± 11.60 | 14.75 ± 13.42  20.14 ± 15.47 | 6.637 | 1 | **0.013** | 0.109 | 70.26 | 1 | **<0.001** | 0.565 | 0.003 | 1 | 0.956 | <0.001 |
| BBT unaffected limb  T0  T2 | 31.86 ± 10.56  40.07 ± 10.36 | 29.39 ± 12.09  32.61 ± 11.14 | 7.647 | 1 | **0.008** | 0.124 | 39.95 | 1 | **<0.001** | 0.425 | 3.111 | 1 | 0.083 | 0.054 |
| BBT score ratio  T0  T2 | 0.40 ± 0.26  0.56 ± 0.24 | 0.56 ± 0.60  0.62 ± 0.46 | 2.685 | 1 | 0.107 | 0.048 | 12.78 | 1 | **0.001** | 0.194 | 1.103 | 1 | 0.298 | 0.020 |
| NPRS  T0  T2 | 3.74 ± 2.89  1.47 ± 1.84 | 1.69 ± 2.45  1.44 ± 2.34 | 8.450 | 1 | **0.005** | 0.123 | 13.03 | 1 | **0.001** | 0.178 | 4.263 | 1 | 0.043 | 0.066 |
| MAS Wrist (a)  T0  T2 | 0 (0-1)  0 (0-1) | 0 (0-1)  0 (0-1) | 2.601 | 1 | 0.107 | - | 97.0 | 1 | 0.186 | - | 0.126 | 1 | 0.723 | - |
| MAS Fingers (a)  T0  T2 | 0 (0-1)  0 (0-1) | 0 (0-1)  0 (0-1) | 2.729 | 1 | 0.099 | - | 13.5 | 1 | **0.004** | - | 0.516 | 1 | 0.473 | - |
| MQoL  T0  T2 | 6.60 ± 1.43  7.32 ± 1.36 | 6.89 ± 1.32  7.37 ± 1.06 | 0.495 | 1 | 0.484 | 0.008 | 13.224 | 1 | **0.001** | 0.176 | 0.373 | 1 | 0.544 | 0.006 |
