# Supplementary material for: Hand rehabilitation with sonification techniques in the subacute stage of stroke
Source: Sci Rep. 2021 Mar 31;11:7237. doi: 10.1038/s41598-021-86627-y (PMC8012636; doi:10.1038/s41598-021-86627-y)
Supplement: Supplementary file 2 — Supplementary Information 2. [file 41598_2021_86627_MOESM2_ESM.docx]

**Supplementary Table S2.** Upper Extremity Scores (Primary and secondary outcomes) at T0, T1, T2 and T3 of the per protocol analysis. Values for continuous variables are

reported as mean ± standard deviation, those for categorical data are reported as median and interquartile range. SG= Sonification Group; SoCG= Standard of Care Group; FM

UE= Fugl-Meyer Upper Extremity scale; BBT= Box and Block Test; NPRS= Numerical Pain Rating Scale; MAS= Modified Ashworth Scale; MQoL-it= McGill Quality of Life

(Italian Version); df=degrees of freedom; (a)=non-parametric (Kruskall-Wallis) statistics.

|  | SG  (n=13) | SoCG  (n=16) | Time*group interaction | | | Effect Size | Time effect | | | Effect Size | Group effect | | | Effect Size |
| --- | --- | --- | --- | --- | --- | --- | --- | --- | --- | --- | --- | --- | --- | --- |
| **Primary Outcomes** |  |  | F | df | P-value |  | F | df | P-value |  | F | df | P-value |  |
| FM-UE Total Score  T0  T1  T2  T3 | 41.85 ± 10.03  50.69 ± 8.82  56.00 ± 8.28  56.08 ± 6.42 | 38.75 ± 17.55  44.00 ± 18.50  48.25 ± 20.05  49.00 ± 19.99 | 1.016 | 3 | 0.402 | 0.109 | 17.45 | 3 | **<0.001** | 0.677 | 1.269 | 1 | 0.270 | 0.045 |
| **Secondary Outcomes** |  |  |  |  |  |  |  |  |  |  |  |  |  |  |
| FM-UE Proximal Score  T0  T1  T2  T3 | 24.69 ± 5.45  29.08 ± 3.86  31.23 ± 4.23  31.23 ± 3.61 | 21.69 ± 10.28  24.94 ± 10.87  27.25 ± 10.82  27.75 ± 10.76 | 0.336 | 3 | 0.800 | 0.039 | 9.87 | 3 | **<0.001** | 0.542 | 1.540 | 1 | 0.225 | 0.054 |
| FM-UE Distal Score  T0  T1  T2  T3 | 12.85 ± 4.81  17.31 ± 5.19  20.15 ± 4.24  20.54 ± 3.71 | 12.56 ± 7.32  14.37 ± 7.91  16.44 ± 9.01  16.69 ± 9.14 | 2.897 | 3 | 0.055 | 0.258 | 24.78 | 3 | **<0.001** | 0.748 | 1.179 | 1 | 0.287 | 0.042 |
| FM-UE Wrist Score  T0  T1  T2  T3 | 5.38 ± 2.29  6.77 ± 2.80  7.85 ± 2.27  8.00 ± 1.83 | 5.00 ± 3.05  5.81 ± 3.23  6.63 ± 3.72  6.94 ± 3.82 | 0.566 | 3 | 0.643 | 0.064 | 14.37 | 3 | **<0.001** | 0.633 | 0.739 | 1 | 0.398 | 0.027 |
| FM-UE Hand Score  T0  T1  T2  T3 | 7.46 ± 3.57  10.54 ± 2.90  12.31 ± 2.46  12.54 ± 2.47 | 7.56 ± 4.46  8.56 ± 4.88  9.81 ± 5.37  9.75 ± 5.42 | 3.424 | 3 | **0.033** | 0.291 | 21.30 | 3 | **<0.001** | 0.719 | 1.438 | 1 | 0.241 | 0.051 |
| BBT affected limb  T0  T1  T2  T3 | 12.69 ± 8.10  19.61 ± 7.87  25.61 ± 8.75  28.23 ± 9.37 | 13.36 ± 10.54  16.36 ± 13.16  20.64 ± 14.78  22.00 ± 15.59 | 1.292 | 3 | 0.301 | 0.144 | 18.01 | 3 | **<0.001** | 0.701 | 0.710 | 1 | 0.407 | 0.028 |
| BBT unaffected limb  T0  T1  T2  T3 | 33.46 ± 10.88  36.85 ± 9.89  42.23 ± 9.41  43.08 ± 11.03 | 30.60 ± 11.85  31.60 ± 10.72  33.20 ± 10.55  34.20 ± 12.17 | 5.715 | 3 | **0.004** | 0.417 | 21.06 | 3 | **<0.001** | 0.725 | 2.744 | 1 | 0.110 | 0.095 |
| BBT score ratio  T0  T1  T2  T3 | 0.38 ± 0.26  0.55 ± 0.21  0.62 ± 0.22  0.67 ± 0.20 | 0.43 ± 0.34  0.48 ± 0.33  0.59 ± 0.37  0.60 ± 0.36 | 0.752 | 3 | 0.532 | 0.089 | 8.28 | 3 | **0.001** | 0.519 | 0.076 | 1 | 0.785 | 0.003 |
| NPRS  T0  T1  T2  T3 | 3.38 ± 2.78  2.18 ± 1.83  1.73 ± 2.00  1.88 ± 2.06 | 1.24 ± 1.75  1.70 ± 2.07  0.85 ± 1.55  1.15 ± 1.66 | 1.145 | 3 | 0.351 | 0.125 | 2.41 | 3 | 0.092 | 0.232 | 4.250 | 1 | **0.049** | 0.140 |
| MAS Wrist (a)  T0  T1  T2  T3 | 0.5 (1)  0 (0.25)  0 (1)  0 (0.25) | 1 (1)  1 (1)  0.5 (1.12)  0 (1.12) | 1.653 | 3 | 0.199 | - | 5.226 | 3 | 0.137 | - | 0.686 | 1 | 0.408 | - |
| MAS Fingers (a)  T0  T1  T2  T3 | 1 (1.12)  0 (1)  0 (1)  0 (0) | 1 (1)  1 (1)  0 (1)  0 (1) | 3.40 | 3 | 0.065 | - | 13.07 | 3 | **0.004** | - | 0.091 | 1 | 0.762 | - |
| MQoL  T0  T2  T3 | 6.29 ± 1.67  7.21 ± 1.58  6.85 ± 1.81 | 6.81 ± 1.44  7.37 ± 1.03  7.34 ± 1.24 | 0.354 | 2 | 0.705 | 0.027 | 4.30 | 2 | 0.024 | 0.249 | 0.722 | 1 | 0.403 | 0.026 |
